# Purification and characterization of a novel ginsenoside Rc-hydrolyzing β-glucosidase from *Armillaria mellea* mycelia

**DOI:** 10.1186/s13568-016-0277-x

**Published:** 2016-11-11

**Authors:** Jitendra Upadhyaya, Min-Sun Yoon, Min-Ji Kim, Nam-Soo Ryu, Young-Eun Song, Young-Hoi Kim, Myung-Kon Kim

**Affiliations:** 1 Department of Food Science and Technology, Chonbuk National University, Jeonju, 54896 Republic of Korea; 2 Department of Food Science and Biotechnology, Chonbuk National University, Iksan, 54596 Republic of Korea; 3 Agricultural Research and Extension Services, Iksan, 54591 Republic of Korea

**Keywords:** *Armillaria mellea*, β-Glucosidase, Enzymatic hydrolysis, Ginsenoside Rc, Minor ginsenosides

## Abstract

Ginsenosides are the principal compounds responsible for the pharmacological effects and health benefits of *Panax ginseng* root. Among protopanaxadiol (PPD)-type ginsenosides, minor ginsenosides such as ginsenoside (G)-F2, G-Rh2, compound (C)-Mc1, C-Mc, C-O, C-Y, and C-K are known to be more pharmacologically active constituents than major ginsenosides such as G-Rb1, G-Rb2, G-Rc, and G-Rd. A novel ginsenoside Rc-hydrolyzing β-glucosidase (BG-1) from *Armillaria mellea* mycelia was purified as a single protein band with molecular weight of 121.5 kDa on SDS-PAGE and a specific activity of 17.9 U mg^−1^ protein. BG-1 concurrently hydrolyzed α-(1 → 6)-arabinofuranosidic linkage at the C-20 site or outer β-(1 → 2)-glucosidic linkage at the C-3 site of G-Rc to produce G-Rd and C-Mc1, respectively. The enzyme also hydrolyzed outer and inner glucosidic linkages at the C-3 site of G-Rd to produce C-K via G-F2, and inner glucosidic linkage at the C-3 site of C-Mc1 to produce C-Mc. C-Mc was also slowly hydrolyzed α-(1 → 6)-arabinofuranosidic linkage at the C-20 site to produce C-K with reaction time prolongation. Finally, the pathways for formation of C-Mc and C-K from G-Rc by BG-1 were G-Rc → C-Mc1 → C-Mc and G-Rc → G-Rd → G-F2 → C-K, respectively. The optimum reaction conditions for C-Mc and C-K formation from G-Rc by BG-1 were pH 4.0–4.5, temperature 45–60 °C, and reaction time 72–96 h. This is the first report of efficient production of minor ginsenosides, C-Mc and C-K from G-Rc by β-glucosidase purified from *A. mellea* mycelia.

## Introduction

**Fig. 1:**
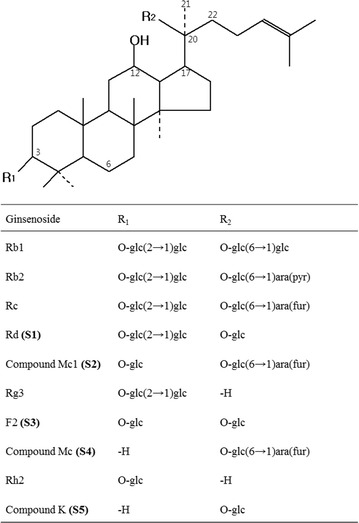
Chemical structures of protopanaxadiol type ginsenosides. The ginsenosides represented are all (*S*)-type ginsenosides. glc, β-d-glucopyranosyl; ara (pyr), α-l-arabinopyranosyl; ara (fur), α-l-arabinofuranosyl

Ginseng, root of *Panax ginseng* C. A. Meyer, has been used as a traditional folk medicine in East Asian countries such as Korea, Japan and China for thousands of years, and has to some extent been popularized in many western countries during recent decades. The major pharmacologically active constituents of ginseng are triterpenoid saponins called ginsenosides. They can be classified into two groups by the skeleton of their aglycones, dammarane-type and oleanane-type. The dammarane-type ginsenosides can also be classified into protopanaxadiol (PPD)-type and protopanaxatriol (PPT)-type (Attele et al. 1999). Naturally occurring major PPD-type ginsenosides such as ginsenoside (G)-Rb1, G-Rb2, G-Rc, and G-Rd (Fig. 1) are hardly absorbed by the human intestinal tract (Hasegawa et al. 1996; Tawab et al. 2003). Conversely, minor ginsenosides such as G-Rg3, G-F2, G-Rh2, and compound (C)-O, C-Y, C-Mc1, C-Mc, and C-K, the hydrolyzed products obtained from major ginsenosides, are more readily absorbed into the bloodstream and function as active compounds (Tawab et al. 2003; Yang et al. 2015). The minor ginsenosides have been demonstrated to possess multiple pharmacological effects, such as anticarcinogenic (Park et al. 2005), immunomodulatory (Liu et al. 2014), anti-inflammatory (Park et al. 2012; Lee and Lau 2011), antiatherosclerotic (Park et al. 2005), antihypertensive (Christensen 2008), antigenotoxic (Lee et al. 1998), and antidiabetic properties (Li et al. 2012). C-K is the major active metabolite of PPD-type ginsenosides produced by human intestinal bacteria (Karikura et al. 1991; Hasegawa et al. 1997).

Various methods have been studied to produce the more active minor ginsenosides from major ginsenosides, including mild acid hydrolysis (Han et al. 1982), alkaline cleavage (Chen et al. 1987), microbial transformation (Bae et al. 2002; Chi and Ji 2005), and enzymatic transformation (Park et al. 2010; Yang et al. 2015). However, the chemical methods such as mild acid hydrolysis and alkaline cleavage result in undesirable side reactions, such as epimerization, hydration, hydroxylation, and random hydrolysis of glycosidic linkages (Wandrey et al. 2000). Although many studies have been carried out to produce minor ginsenosides from major ginsenosides by microbial and enzymatic methods (Park et al. 2010; Yang et al. 2015), some of microbial methods were limited by safety problems for food application and also some of enzymatic biotransformation methods have serious limitation owing to little or no activity toward G-Rc, C-Mc1 which harbor α-l-arabinofuranosyl moiety and low yields (Park et al. 2010).

The fruiting bodies of basidiomycete mushrooms have been used in many cuisines worldwide as food ingredients and some mushrooms have long been used in traditional Chinese medicine (Chen et al. 2015). Mushrooms are fungi of great interest that secrete various hydrolytic enzymes such as cellulase, β-glucosidase, and endo- and exoglucanase (Buswell et al. 1996; Baldrian and Valášková 2008; Mfombep et al. 2013). Previous study (Upadhyaya et al. 2016) reported that C-K was found to be produced with high yield from G-Rb1 by ammonium sulfate (30–80%) precipitate isolated from the cultured mycelia of *Armillaria mellea* (AMMEP). Therefore, we investigated the possibility of producing minor ginsenosides from G-Rc, one of major PPD-type ginsenosides (Fig. 1), by using ammonium sulfate (30–80%) precipitates isolated from the cultured mycelia of five edible and/or medicinal mushrooms. And then we found that AMMEP have a strong hydrolytic activity of G-Rc into the minor ginsenosides, C-Mc and C-K. C-K has received increasing attention because of its pharmacological activities, for example, anti-inflammatory, anticarcinogenic, antiangiogenesis, antiaging, antiallergic, antidiabetic, and hepatoprotective effects, whereas relatively little is known about the pharmacological activity of C-Mc, except for its anti-inflammatory activity in vitro (Bae et al. 2002). In this study, β-glucosidase (BG-1) which specifically hydrolyzes G-Rc into C-Mc and C-K was homogeneously purified from *A*. *mellea* mycelia. In addition, the hydrolytic properties of G-Rc by a purified BG-1 were characterized.

## Materials and methods

### Materials

Strains of *A. mellea* (KACC 50013), *Ganoderma lucidum* (KACC 42231), *Phellinus baumii* (KACC 53719), *Ganoderma applanata* (KACC 53688), and *Pleurotus ostreatus* (KACC 50356) were donated by the Korean Agricultural Culture Collection (KACC, Suwon, Gyeonggi-Do, Republic of Korea). G-Rc was isolated from the total crude ginseng saponin fraction according to the reported procedure (Sanada et al. 1974). The purified compound was identified by comparison of spectral data and retention time by HPLC with that of an authentic sample. Authentic standard mixture of G-Rb1, Rb2, G-Rc, G-Rd, G-Rg3 (*S*), G-F2, C-K, C-Mc1 and C-Mc were freely obtained from the Korea Ginseng Corporation Research Institute (Daejeon, Republic of Korea). *p*-Nitrophenol, *p*-nitrophenyl (*p*NP)-β-d-glucopyranoside, *p*NP-α-d-glucopyranoside, *p*NP-β-d-galactopyranoside, *p*NP-α-d-galactopyranoside, *p*NP-α-l-arabinofuranoside, *p*NP-α-l-arabinopyranoside, DEAE cellulose, Sephadex G-150, bovine serum albumin (BSA) and 4-methylumbelliferyl-β-d-glucopyranoside (MUG) were purchased from Sigma-Aldrich Co. (St. Louis, MO, USA). Mini-protean TGX precast gel and prestaind protein standards for SDS-PAGE was purchased from Bio-RAD (Hercules, CA, USA). Diaion HP-20 resin (250–850 µm) was purchased from Supelco Co. (Bellefonte, PA, USA). Silica gel 60 F_254_ TLC plates and silica gel 60 (230–400 mesh) for column chromatography were purchased from Merck Co. (Darmstadt, Germany). Other reagents were of analytical reagent grade from commercial sources.

### Cultivation of mushroom mycelia

Mushroom mycelia were cultivated following the procedure described in our previous paper (Upadhyaya et al. 2016). Strains were pre-incubated on potato dextrose agar (Becton, Dickinson and Company, Sparks, MD, USA) for 6 days at 25 °C. All nutrient media were sterilized at 121 °C for 30 min. The pre-incubated strain was inoculated into germinated-malt medium (11 Brix°) saccharified at 65 °C with fourfold tap water (v/v) for 8 h, then cultured for 2 weeks at 25 °C. Scaled-up production of mushroom mycelia except for *A. mellea* was performed in 4 l Erlenmeyer flasks containing 1 l of germinated-malt medium (11 Brix°) for 3 weeks at 25–26 °C with gentle shaking (120 rpm). Standing liquid culture of *A. mellea* mycelium was performed at 25–26 °C for 3 weeks in polypropylene bottles (1.2 l) for mushroom cultivation filled with 800 ml of saccharified malt medium (11 Brix°).

### Preparation of crude enzymes

All procedure for enzyme purification was carried out at room temperature unless otherwise indicated. Culture media was filtered through cheese cloth to separate the mycelia from the broth. The mycelial mass was washed with distilled water to remove residual broth, and then lyophilized. Fifty gram of each lyophilized mycelial mass was mixed with 500 ml of 0.1 M sodium phosphate buffer (pH 4.8) with gentle stirring for 12 h at 4 °C, then homogenized with an Omni mixer homogenizer (Omni International, Kennesaw, GA, USA) for 1 min at 4 °C. The slurry was squeezed through cheese cloth and the filtrate was centrifuged at 10,000×*g* for 20 min at 4 °C. Solid ammonium sulfate was added to the supernatant (400 ml), initially to 30% and eventually to 80% saturation. After centrifugation at 10,000×*g* for 20 min at 4 °C, the precipitate was dissolved in 10 mM sodium acetate buffer (pH 4.8). After overnight dialysis, the solution was centrifuged at 10,000×*g* for 20 min at 4 °C, and the supernatant was lyophilized.

### Purification of β-glucosidase (BG-1) from *A. mellea* mycelia

One hundred gram of lyophilized mycelial mass of *A. mellea* was mixed with 1 l of 0.1 M acetate buffer (pH 4.8) with gentle stirring for 12 h at 4 °C, then homogenized with an Omni mixer homogenizer for 1 min at 4 °C. The slurry was squeezed through cheese cloth and the filtrate was centrifuged at 10,000×*g* for 20 min at 4 °C. Solid ammonium sulfate was added to the supernatant, initially to 30% and eventually to 80% saturation. After centrifugation at 10,000×*g* for 20 min at 4 °C, the precipitate was dissolved in 10 mM sodium acetate buffer (pH 4.8). The concentrated extract (30 ml) was loaded onto a DEAE cellulose column (18 cm × 3.0 cm) pre-equilibrated with 10 mM acetate buffer (pH 4.8). The column was washed with same buffer for 20 min, and thereafter the bound proteins were eluted with linear gradient condition of 0–0.5 M NaCl at a flow rate of 1 ml min^−1^, and fractionated into 3.0 ml per tube. The active fractions were pooled, dialyzed against 10 mM acetate buffer (pH 4.8) using 14 kDa cut-off dialysis tube (Viskase Co., Lombard, IL, USA), and was concentrated by lyophilization. The concentrate was applied to Sephadex G-150 column (70 cm × 1.8 cm) pre-equilibrated with 10 mM acetate buffer (pH 4.8) and the fraction were collected at a flow rate of 0.4 ml min^−1^. The fraction containing β-glucosidase activity was pooled and lyophilized for further characterization.

### Electrophoretic analysis

Sodium dodecyl sulfate–polyacrylamide gel electrophoresis (SDS-PAGE) was performed with a 12% mini-protean TGX precast gel at a constant current of 110 mA according to the method of Laemmli (1970). The gel was stained with Coomassie brilliant blue R-250 and destained with a mixture of 10% methanol and 10% acetic acid in distilled water. Native PAGE was performed with a 12% mini-protean TGX precast gel under the above condition. After electrophoresis, the gel was immersed in 0.1 M acetate buffer (pH 4.8) containing 0.1% 4-methylumbelliferyl-β-d-glucopyranoside (MUG) as a substrate for 30 min at 37 °C. The aglycone liberated was detected under ultraviolet (UV) light (365 nm).

### Enzyme assays

β-Glucosidase activity was assayed as described by Mfombep et al. (2013) with some modifications. Briefly, the reaction mixture (1.0 ml), containing 0.1 ml of *p*NP-β-d-glucopyranoside (10 mM), 0.1 ml of appropriately diluted enzyme solution, and 0.8 ml of 0.1 M acetate buffer (pH 4.8), was incubated for 30 min at 37 °C. The reaction was terminated by adding 1.0 ml of 0.5 M Na_2_CO_3_ solution. The released *p*-nitrophenol was measured immediately using a UV–visible spectrophotometer (UV-1601, Shimadzu, Tokyo, Japan) at 400 nm. Activities toward other *p*NP glycosides were assayed in the same way. The amount of *p*-nitrophenol released was quantified using a concentration plot of a *p*-nitrophenol standard. One unit of enzyme activities were defined as the amount of enzyme required to release 1 μM of *p*-nitrophenol min^−1^ under the assay conditions.

### Enzymatic hydrolysis of G-Rc and enzyme characterization

The reaction mixture (2.0 ml) containing 2.0 mg of G-Rb1, G-Rc or G-Rd in 0.2 ml of methanol and each enzyme solution showing 1.5 U of β-glucosidase activity in 1.8 ml of 0.1 M sodium acetate buffer (pH 4.8) were incubated for 96 h at 45 °C, respectively. The reaction mixture was extracted twice with 2.0 ml of water saturated *n*-butanol. The *n*-butanol fraction was concentrated to dryness in vacuo, and the residue was dissolved in 1.0 ml of methanol. To investigate the time course of G-Rc hydrolysis by β-glucosidase (BG-1) purified from *A. mellea* mycelia, 20 ml of reaction mixture containing 20 mg of G-Rc in 2.0 ml methanol, enzyme solution containing 15 U of BG-1 and 0.1 M sodium acetate buffer (pH 4.8) was incubated for 96 h at 45 °C with gentle shaking. Two milliliters of the reaction mixture were withdrawn at regular time intervals, and extracted twice with 2.0 ml of water saturated *n*-butanol. The *n*-butanol fraction was concentrated to dryness in vacuo. The residue was dissolved in 1.0 ml of methanol, and was subjected to TLC and HPLC analysis.

The effect of temperature on hydrolytic activity of G-Rc was examined by incubating the reaction mixture at temperatures ranging from 30 to 70 °C for 96 h at pH 4.8. The effect of pH was examined using G-Rc as a substrate for 96 h at 45 °C in the following buffer solutions (each at 0.1 M): glycine–HCl (pH 3.0), sodium acetate (pH 4.0, 4.5, 5.0, 5.5), sodium phosphate (pH 6.0 and 7.0), Tris–HCl (pH 8.0), and glycine-NaOH (pH 9.0). To investigate the effect of enzyme concentration, the enzyme solutions containing β-glucosidase activity ranging from 0.1 to 1.6 U in 1.8 ml of 0.1 M sodium acetate buffer (pH 4.8) were incubated with 2.0 mg of G-Rc in 0.2 ml of methanol for 96 h at 45 °C. The relative ratio of hydrolysis products in the reaction mixtures were calculated from the peak area percentages in HPLC analysis without consideration of the detector response factor. All experiments were performed in triplicate, and the data are expressed as the mean ± standard deviation (SD).

### Isolation and identification of hydrolysis products

A reaction mixture containing 0.6 g of G-Rc in 10 ml of methanol, enzyme solution containing 450 U of BG-1, and 290 ml of 0.1 M sodium acetate buffer (pH 4.8), making the final volume of 300 ml, was incubated for 24 h at 45 °C with gentle stirring. After a 10 min heat-treatment in boiling water, the reaction mixture was passed through a Diaion HP-20 column (40 cm × 4 cm) at a flow rate of 4 ml min^−1^. The resin was washed with 500 ml of distilled water to remove water soluble sugars. The hydrolysis products were eluted from the resin with 400 ml of methanol. The eluate was then concentrated to dryness in vacuo. The concentrate was chromatographed on a silica gel column using stepwise gradient elution with chloroform–methanol-water (90:10:0.5 → 80:20:2 → 60:35:10, v/v/v, lower phase). The yields of metabolites S1, S2, S3, S4, and S5 were 35, 23, 18, 24, and 23 mg, respectively.

### TLC analysis

TLC was performed on silica gel 60 F_254_ with chloroform–methanol-water (65:35:10, v/v/v, lower phase) as the developing solvent. The spots on the TLC were detected by spraying 10 % (w/v) sulfuric acid in ethanol, followed by heating at 110 °C for 10 min.

### HPLC and UPLC/Q-TOF–MS analysis

HPLC analysis was performed using an HPLC system (Waters, Milford, MA, USA) equipped with a 600E system controller, 717 plus autosampler and 486 UV detector (203 nm) with a YMC C_18_ column (250 mm × 4.6 mm, 5 µm, YMC Co. Ltd., Tokyo, Japan). The mobile phase consisted of water (A) and acetonitrile (B) at ratios of A:B 70:30 (0–15 min), 43:57 (15–25 min), 30:70 (25–30 min), and 70:30 (30–35 min) at a flow rate of 0.9 ml min^−1^. UPLC/Q-TOF–MS analysis was performed using a Waters ACQUITY UPLC system composed of a binary solvent manager and a photo diode array detector (203 nm). The chromatographic separation was performed on an ACQUITY UPLC BEH C_18_ column (100 mm × 2.1 mm, 1.7 µm). The column temperature was 40 °C. The binary gradient elution system consisted of 0.001% phosphoric acid in water (A) and 0.001% phosphoric acid in acetonitrile (B). The separation was achieved using the following gradient program of A:B = 85:15 (0–0.5 min), 70:30 (14.5 min), 68:32 (15.5 min), 62:38 (18.5 min), 57:43 (24.0 min), 45:55 (31.0 min), 30:70 (35.0 min), 10:90 (38.0 min), 85:15 (43.0 min) (Park et al. 2013). The flow rate was 0.6 ml min^−1^. MS analysis was performed on a Waters Xevo quadruple-time of flight mass spectrometer (Q-TOF–MS) equipped with an electrospray ionization (ESI) source in negative ion mode. The conditions for MS analysis were: drying gas N_2_, flow rate 12 l min^−1^, cone gas temperature 350 °C, nebulizer pressure 50 psi, and capillary voltage 4.0 kV.

### NMR analysis

NMR spectra were taken on a JEOL model JNM-ECA 600 FT-NMR spectrometer (Akishima, Tokyo, Japan) at 600 MHz (^1^H NMR) and 150 MHz (^13^C NMR) in pyridine-d_5_ with tetramethylsilane as an internal standard.

## Results

### Purification of β-glucosidase from *A. mellea* mycelia

**Fig. 2:**
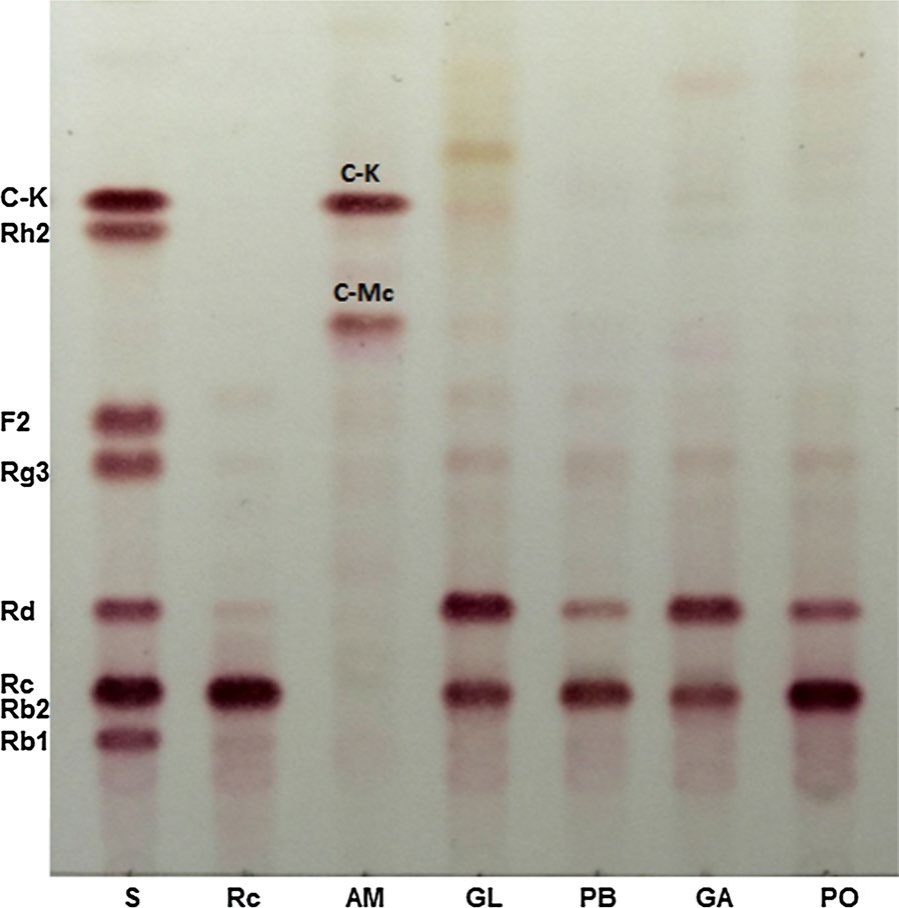
TLC analysis of hydrolysis products of G-Rc by ammonium sulfate (30–80%) precipitates isolated from mushroom mycelia. S, Mixture of authentic ginsenosides; Rc, G-Rc; AM, *Armillaria mellea*; GL, *Ganoderma lucidum*; PL, *Phellinus baumii*; GA, *Ganoderma applanata*; PL, *Pleurotus ostreatus*

Five edible and/or medicinal mushrooms were screened for their ability to hydrolyze G-Rc into minor ginsenosides. The result showed that C-Mc and C-K were efficiently produced from G-Rc by crude enzyme preparations from *A. mellea* mycelia, whereas crude enzyme preparations from *G. lucidum*, *P. baumii*, *G. applanata*, and *P. ostreatus* produced of G-Rd as a final product (Fig. 2). *Armillaria mellea* mycelia has potential to be used to prepare minor ginsenosides such as C-Mc and C-K with high yield from G-Rc, and was chosen for further study.

**Fig. 3:**
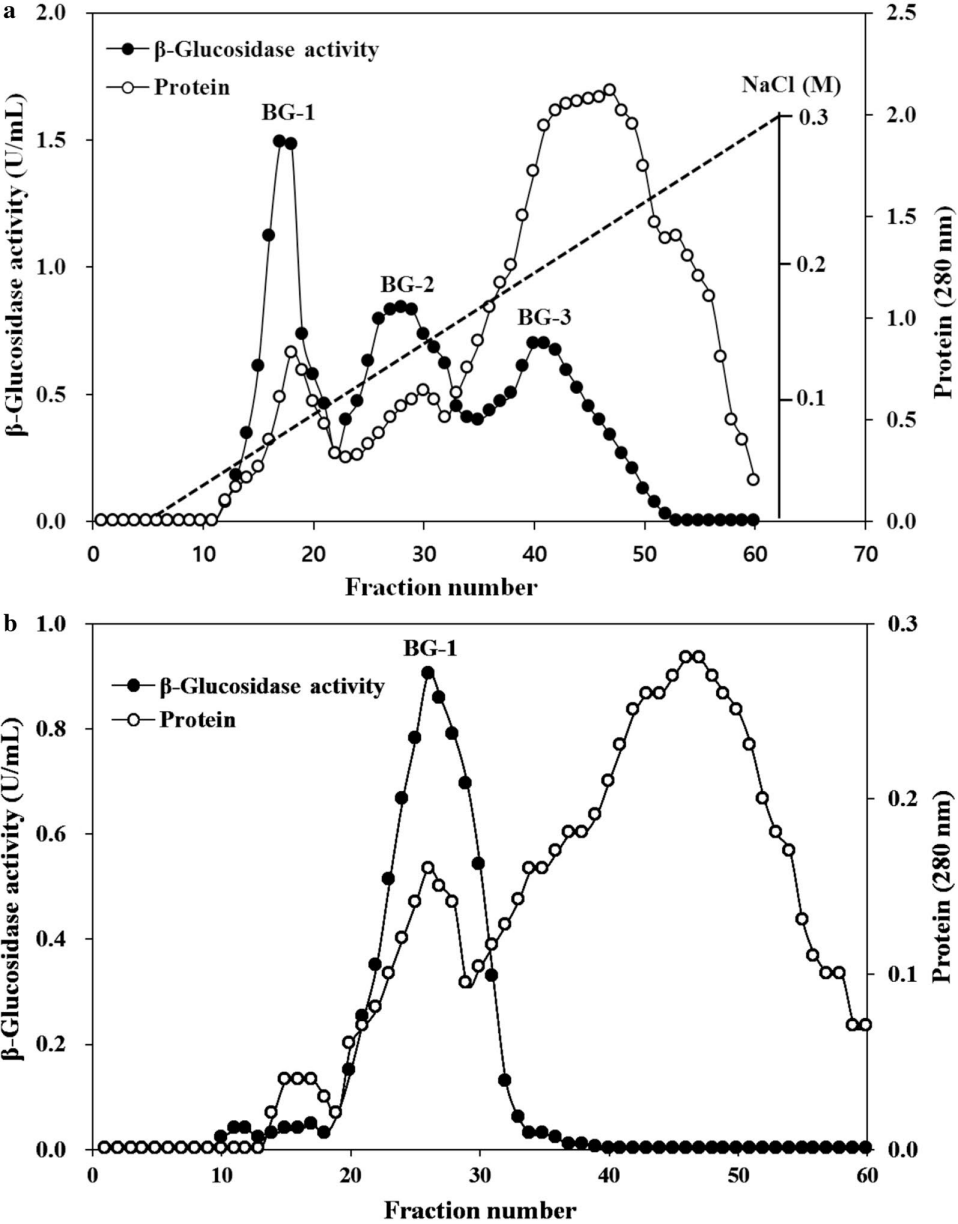
**a** DEAE cellulose ion exchange chromatography. **b** Sephadex G-150 gel chromatography

**Fig. 4:**
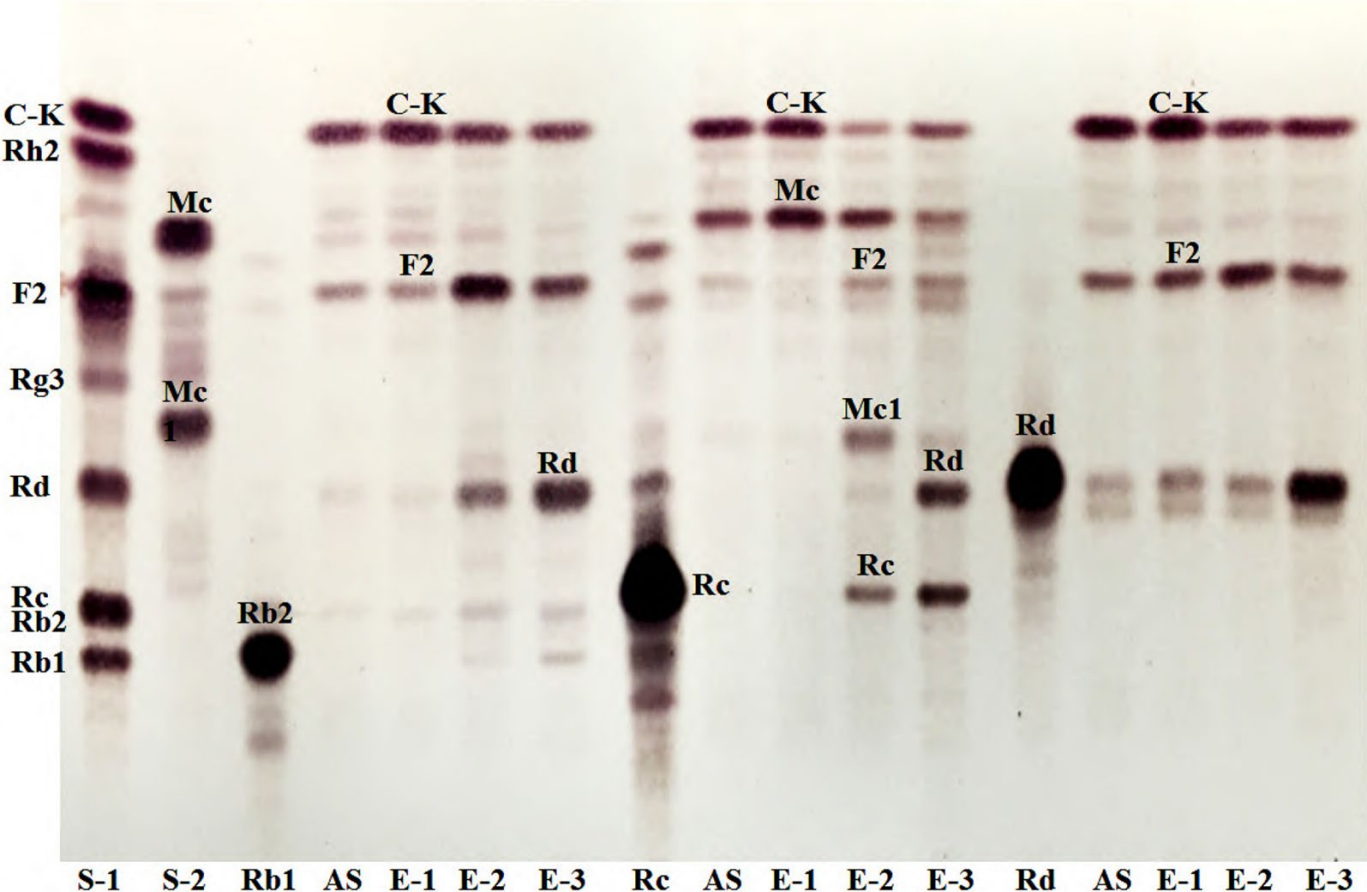
TLC analysis of hydrolysis products of G-Rb1, G-Rc and G-Rd by AS (30–80% ammonium sulfate precipitate); *E-1* (BG-1); *E-2* (BG-2); *E-3* (BG-3)

**Fig. 5:**
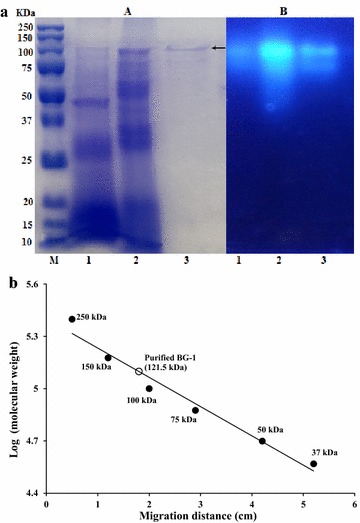
**a** SDS-PAGE (*A*) and native PAGE (*B*) analysis of the purified BG-1 enzyme. *M* protein molecular weight marker; *lane 1* ammonium sulfate precipitate; *lane 2* DEAE cellulose ion exchange chromatography; *lane 3* purified BG-1. **b** Determination of molecular mass of the purified BG-1 by SDS-PAGE

β-Glucosidases in ammonium sulfate precipitate isolated from *A. mellea* mycelia were eluted as three active fractions by DEAE cellulose ion exchange chromatography (Fig. 3a). These enzymes in relevant fraction were designated as BG-1, BG-2 and BG-3, respectively. The results indicate that *A. mellea* β-glucosidases exist in three isomeric forms that they exhibited different retention behaviors on DEAE cellulose column and different hydrolytic activity toward *p*NP-β-d-glucopyranoside. BG-1 was further purified by Sephadex G-150 gel chromatography (Fig. 3b). Finally, the BG-1 was purified approximately 34 fold with a yield of 1.44% relative to the crude enzyme extract. When G-Rb1, G-Rc and G-Rd were used as the substrates, BG-1 showed different hydrolytic patterns from those of BG-2 and BG-3 with potent hydrolytic activity toward G-Rc (Fig. 4). The specific activity of the purified enzyme was 17.9 U mg^−1^ protein. BG-1 was purified to homogeneity as shown by both SDS-PAGE and native PAGE (Fig. 5a). Compared with protein markers, the molecular weight of BG-1 was estimated as 121.5 kDa on SDS-PAGE (Fig. 5b). A summary of the purification result is shown in Table 1. When substrate specificities of BG-1 were as assayed using of *p*NP-glycosides with α- and β-configurations. BG-1 showed hydrolytic activities toward *p*NP-α-l-arabinofurnoside and *p*NP-α-l-arabinopyranoside besides *p*NP-β-d-glucopyranoside, but not toward *p*NP-α-d-glucopyranoside and *p*NP-α- and -β-galactopyranoside (Table 2).

**Table 1:** Purification of BG-1 from *A. mellea* mycelia

	Total protein (mg)	Total activity (U)	Specific activity (U mg^−1^)	Yield (%)	Purification (fold)
Crude extract	1119.4	595.5	0.53	100	1
30–80% (NH_4_)_2_SO_4_	154.7	178.6	1.15	30.0	2.17
DEAE cellulose	10.6	51.8	4.89	8.70	9.23
Sephadex G-150	0.48	8.60	17.9	1.44	33.8

**Table 2:** Relative activity of BG-1 on various chromogenic substrates

Substrate	Activity (%)
*p*NP-β-d-glucopyranoside	100
*p*NP-α-d-glucopyranoside	0
*p*NP-α-l-arabinopyranoside	10.2
*p*NP-α-l-arabinofuranoside	30.0
*p*NP-β-d-galactopyranoside	0
*p*NP-α-d-galactopyranoside	0

Relative activity expressed relative to activity on

*p*NP-β-d-glucopyranoside (100%)

### Isolation and identification of hydrolysis products

**Fig. 6:**
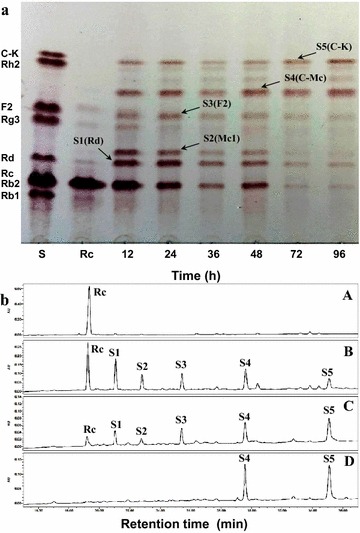
**a** TLC analysis of the reaction mixture during hydrolysis of G-Rc by BG-1. **b** HPLC analysis of the reaction mixture. *A* control (0 h); *B* 24 h; *C* 48 h; *D* 96 h

**Fig. 7:**
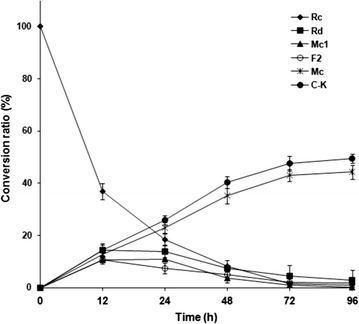
Time course of hydrolysis of G-Rc by BG-1

To investigate the hydrolysis pattern of G-Rc by BG-1 with reaction time, the reaction mixture was withdrawn at regular time intervals during the enzymatic hydrolysis. TLC (Fig. 6a) and HPLC (Fig. 6b) profiles showed that G-Rc was gradually hydrolyzed to five compounds (hydrolysis products S1, S2, S3, S4, and S5). G-Rc was hydrolyzed into S1 and S2 in the early stage of the reaction (within 24 h). After 96 h reaction, almost all of the G-Rc and products S1, S2 and S3 were hydrolyzed into products S4 and S5 (Fig. 7). These new spots were not observed when the reaction mixture containing only G-Rc was incubated in for 96 h at 45 °C and a 10 min heat-treatment in boiling water. These results suggest that products S1, S2, and S3 were intermediate products, while S4 and S5 were final hydrolysis products. The mixture after 24 h reaction was analyzed by UPLC/Q-TOF–MS, and the products were isolated in a pure state by repeated silica gel column chromatography to determine their chemical structures.

Product S1 appeared as a quasi-molecular ion peak at *m/z* 991.5038 [M–H + HCOOH]^−^ with a molecular ion peak [M–H]^−^ at *m/z* 945.5298 by Q-TOF-LC/MS analysis, corresponding to the molecular formula C_48_H_82_O_18_ (MW 946.5501). The ^1^H-NMR spectrum of S1 showed eight methyl signals assignable to an aglycone part at δ_H_ (C_5_D_5_N) 0.80, 0.94, 0.96, 1.11, 1.28, 1.60, 1.60, and 1.63 (all 3H, all s), and anomeric proton signals due to three β-glucosidic linkages at δ_H_ 4.93 (1H, d, *J* = 7.42 Hz, H-1 of inner glucose at C-3 of aglycone), δ_H_ 5.15 (1H, d, *J* = 7.60 Hz, H-1 of glucose at C-20 of aglycone), and δ_H_ 5.39 (1H, d, *J* = 7.56 Hz, H-1 of outer glucose at C-3 of aglycone). The ^13^C-NMR spectrum of S1 showed three anomeric carbon signals at δ_C_ 98.1, 105.0, and 105.9 with 30 carbon signals ascribable to aglycone and 18 carbon signals ascribable to three glucoses (Table 3). Accordingly, S1 was determined to be 3-*O*-[β-d-glucopyranosyl-(1 → 2)-β-d-glucopyranosyl]-20-*O*-β-d-glucopyranosyl-20(*S*)-protopanaxadiol (G-Rd).

**Table 3:** ^13^C-NMR chemical shifts of hydrolysis products of G-Rc by BG-1

Carbon no	G-Rd	C-Mc1	G-F2	C-Mc	C-K
Aglycone moiety					
1	39.0	39.6	38.9	39.9	39.8
2	26.6	27.2	26.5	26.6	26.6
3	88.8	89.2	88.6	78.5	78.7
4	39.5	40.1	39.5	39.8	39.9
5	56.2	56.8	56.1	56.6	56.8
6	18.3	18.2	18.2	18.3	19.2
7	35.0	35.5	34.9	35.6	35.6
8	39.9	40.4	39.8	40.0	40.0
9	50.0	50.6	50.0	50.7	50.8
10	36.7	37.3	36.7	37.8	37.8
11	30.6	30.6	30.6	30.6	31.3
12	70.1	70.6	70.1	70.6	70.6
13	49.3	49.8	49.5	49.9	50.0
14	51.4	52.0	51.2	51.8	52.0
15	30.7	30.4	29.8	31.1	31.4
16	26.5	27.0	26.4	26.2	26.3
17	51.5	51.8	51.5	52.1	51.9
18	16.1	16.7	16.1	16.7	16.5
19	15.8	16.4	15.7	16.5	16.8
20	83.2	82.8	83.1	83.8	83.8
21	22.3	22.3	22.3	22.8	22.8
22	35.9	36.5	35.8	36.6	36.7
23	23.1	23.6	23.1	23.6	23.7
24	125.8	126.4	125.7	125.3	126.5
25	130.8	131.3	130.8	131.4	131.4
26	25.7	26.2	25.6	26.2	26.2
27	17.6	17.2	17.6	17.8	17.9
28	27.9	28.5	27.9	27.2	28.8
29	16.5	16.7	16.6	16.8	16.7
30	17.2	17.8	17.1	17.8	17.6
3-Glucopyranosyl (inner)					
1′	105.0	105.4	106.7		
2′	83.2	76.2	75.6		
3′	78.1	79.3	77.8		
4′	71.5	72.3	71.7		
5′	78.1	79.1	78.2		
6′	62.6	63.0	62.8		
3-Glucopyranosyl (outer)					
1″	105.9				
2″	77.0				
3″	79.1				
4″	71.5				
5″	78.1				
6″	62.7				
20-Glucopyranosyl					
1′	98.1	98.5	98.1	98.5	98.7
2′	75.0	75.2	75.0	75.4	75.6
3′	77.8	78.6	79.0	79.7	77.8
4′	71.4	72.1	71.3	72.6	72.1
5′	78.0	76.9	78.5	77.0	78.5
6′	62.5	68.8	62.5	68.9	63.4
20-Arabinofuranosyl					
1″		110.5		110.5	
2″		83.7		83.7	
3″		78.7		78.6	
4″		86.5		86.5	
5″		63.0		83.1	

S2 showed quasi-molecular ion peaks at *m/z* 961.4637 [M–H + HCOOH]^−^ and 915.5251 [M–H]^−^ by UPLC/Q-TOF–MS analysis, corresponding to the molecular formula C_47_H_80_O_17_ (MW 916.5396). The ^1^H-NMR spectrum of S2 showed eight methyl signals assignable to an aglycone part at δ_H_ 0.75, 0.90, 0.92, 0.96, 1.33, 1.56, 1.56, and 1.59 (all 3H, all s), and three anomeric proton signals due to two β-glucosidic linkages at δ_H_ 4.95 (1H, d, *J* = 7.60 Hz) and 5.15 (1H, d, *J* = 7.90 Hz) and one α-arabinofuranosidic linkage at δ_H_ 5.69 (1H, d, *J* = 1.72 Hz, H-1 of outer arabinofuranose at C-20 of aglycone). In ^13^C-NMR chemical shifts (Table 3), three anomeric carbon signals were observed, at δ_C_ 98.5 and 105.0 due to two β-glucosidic linkages, and δ_C_ 110.5 due to one α-arabinofuranosidic linkage. Therefore, product S2 was identified as 3-*O*-β-d-glucopyranosyl-20-*O*-[α-l-arabinofuranosyl-(1-6)-β-d-glucopyranosyl]-20(*S*)-protopanaxadiol (C-Mc1).

S3 showed a quasi-molecular ion peak at *m/z* 829.4857 [M–H + HCOOH]^−^ by UPLC/Q-TOF–MS analysis, corresponding to the molecular formula C_42_H_72_O_13_ (MW 784.4973). The ^1^H-NMR spectrum of S3 showed eight methyl signals assignable to an aglycone part at δ_H_ 0.79, 0.92, 0.94, 0.97, 1.30, 1.58, 1.58, and 1.61 (all 3H, all s) and two anomeric proton signals due to β-glucosidic linkages at δ_H_ 4.93 (1H, d, *J* = 7.02 Hz, glucose at C-3 position of aglycone) and δ_H_ 5.22 (1H, d, *J* = 7.32 Hz, glucose at C-20 position of aglycone). There were two anomeric carbon signals at δ_C_ 98.1 and 106.7 due to β-glucosidic linkages in the ^13^C-NMR spectrum. Therefore, S3 was identified as 3-*O*-β-d-glucopyranosyl-20-*O*-β-d-glucopyranosyl-20(*S*)-protopanaxadiol (G-F2).

S4, one of the two final products, showed quasi-molecular ion peaks at 799.4570 [M–H + HCOOH]^−^ and 753.4780 [M–H]^−^ corresponding to the molecular formula C_41_H_72_O_12_ (MW 754.4788). The ^1^H-NMR spectrum of S4 showed eight methyl signals assignable to an aglycone part at δ_H_ 0.89, 0.94, 1.00, 1.04, 1.23, 1.63, 1.65, and 1.67 (all 3H, all s), and two anomeric proton signals due to one β-glucosidic linkage at δ_H_ 5.15 (1H, d, *J* = 7.60 Hz, H-1 of inner glucose at C-20 of aglycone) and one α-arabinofuranosidic linkage at δ_H_ 5.67 (1H, d, *J* = 1.72 Hz, H-1 of outer arabinofuranose at C-20 of aglycone). In the ^13^C-NMR spectrum, two anomeric carbon signals were observed at δ_C_ 98.5 due to one β-glucosidic linkage and at δ_C_ 110.5 due to one α-arabinofuranosidic linkage. From these results, product S4 was determined to be 20-*O*-[α-l-arabinofuranosyl-(1-6)-β-d-glucopyranosyl]-20(*S*)-protopanaxadiol (C-Mc).

Product S5 showed quasi-molecular ion peaks at *m/z* 667.4309 [M–H + HCOOH]^−^ and 621.4309 [M–H]^−^ by UPLC/Q-TOF–MS analysis, corresponding to the molecular formula C_36_H_62_O_8_ (MW 622.4445). The ^1^H-NMR spectrum of S5 showed eight methyl signals assignable to an aglycone part at δ_H_ 0.89, 0.95, 0.99, 1.04, 1.23, 1.60, 1.60, 1.63 (all 3H, all s), and one anomeric proton signal due to a β-glucosidic linkage at δ_H_ 5.18 (1H, d, *J* = 7.66 Hz, glucose at C-20 position of aglycone). The ^13^C-NMR spectrum of S5 showed one anomeric carbon signal at δ_C_ 98.7 with 30 carbon signals ascribable to aglycone and six carbon signals ascribable to one glucose. The HPLC retention time of S5 was consistent with that of standard C-K. From these results, product S5 was identified as 20-*O*-β-d-glucopyranosyl-20(*S*)-protopanaxadiol (C-K). The ^13^C-NMR chemical shifts for compounds in the present study are consistent with previous results (Bae et al. 2002; Liu et al. 2015).

### Hydrolytic characterization of G-Rc by BG-1

**Fig. 8:**
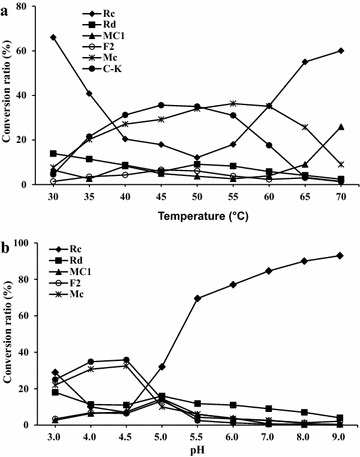
**a** Influence of temperature on hydrolysis of G-Rc by BG-1. **b** Influence of pH on hydrolysis of G-Rc by BG-1

**Fig. 9:**
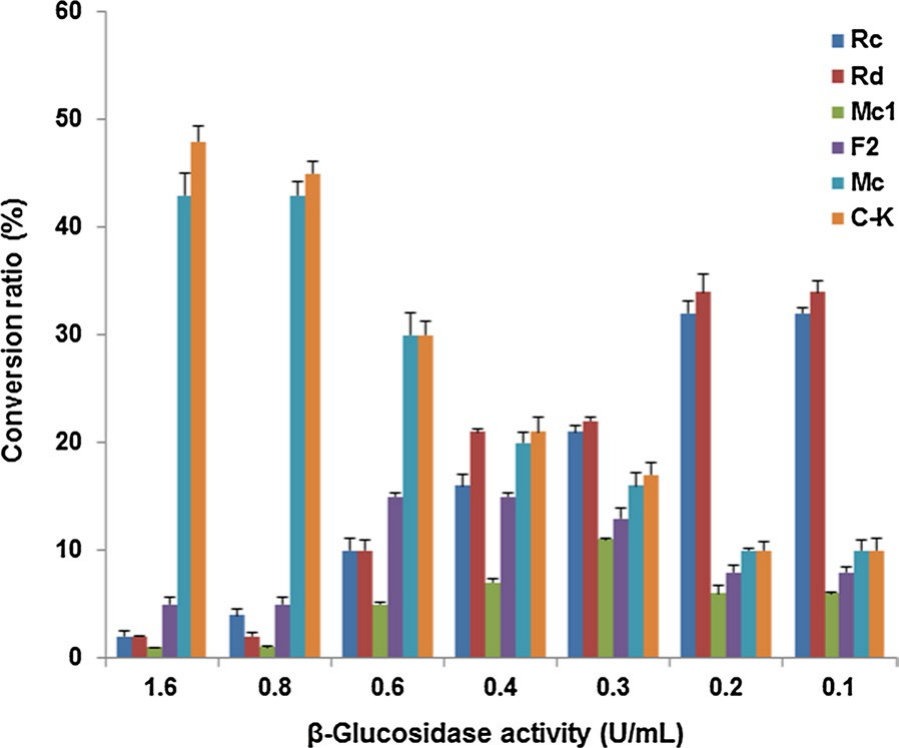
Influence of enzyme concentration on hydrolysis of G-Rc by BG-1

When hydrolysis of G-Rc by BG-1 was conducted at various temperatures, the hydrolysis of G-Rc was maximized at 45–60 °C. Interestingly, the optimum temperature for C-Mc and C-K formation were slightly different, 55–60 °C for C-Mc and 45–50 °C for C-K (Fig. 8a), respectively. Hydrolysis of G-Rc was decreased at temperatures below 35 °C and above 65 °C. To investigate the effect of pH on the hydrolytic activity of G-Rc by BG-1, pH was varied from 3.0 to 9.0 as shown in Fig. 8b. G-Rc was hydrolyzed into G-Rd, C-Mc1, G-F2, C-Mc, and C-K at pH 3.0. C-Mc and C-K formation reached their maxima at pH 4.0–4.5. When the pH value was increased to ≥5.0, the hydrolytic activity of G-Rc was decreased. These results suggest that the optimum pH range for hydrolysis of G-Rc by BG-1 is between pH 4.0 and 4.5. The effect of enzyme concentration on the formation of C-Mc and C-K was examined. As shown in Fig. 9, as the concentration of enzyme in the reaction mixture was increased, the conversion ratio of G-Rc to C-Mc and C-K was increased. When G-Rc (2.0 mg) was incubated with BG-1 containing 0.8–1.6 U of β-glucosidase activity in the reaction mixture (1 ml) for 96 h at 45 °C, G-Rc was completely hydrolyzed to C-Mc and C-K.

### Hydrolysis pathways of G-Rc by BG-1

**Fig. 10:**
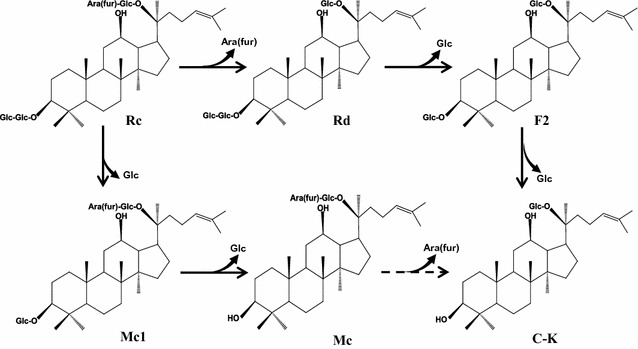
Pathways of C-Mc and C-K formation from G-Rc by BG-1

G-Rc has two glucose moieties at the C-3 position of the PPD-type aglycone, and one arabinofuranose and one glucose at the C-20 position. Therefore, G-Rc can be hydrolyzed by β-glucosidases via multiple pathways. In this study, the results obtained from TLC, HPLC, and UPLC/Q-TOF–MS analysis showed that hydrolysis of G-Rc by BG-1 occurred through two main pathways, as shown in Fig. 10. In one pathway, BG-1 first hydrolyzed the outer α-l-arabinofuranosidic linkage attached to the C-20 position of the aglycone to produce G-Rd, followed by hydrolysis of the outer and inner glucose moieties attached to the C-3 position to produce C-K via G-F2. Concurrently, in the second pathway, BG-1 hydrolyzed the outer β-glucosidic linkage attached to the C-3 position of the G-Rc aglycone to produce C-Mc1, followed by hydrolysis of the inner glucosidic linkage attached to the C-3 position to produce C-Mc.

## Discussion

In the screening for mushroom mycelia containing G-Rc-hydrolyzing activity, we found that enzyme preparation from *A. mellea* mycelia could efficiently convert G-Rc to C-Mc and C-K, whereas enzyme preparations from *G. lucidum*, *P. baumii*, *G. applanata*, and *P. ostreatus* produced of G-Rd as a final product. *Armillaria mellea* β-glucosidase exists in three isomeric forms (BG-1, BG-2 and BG-3) that they exhibited different retention behaviors on DEAE cellulose column and hydrolytic activity toward *p*NP-β-d-glucopyranoside. Moreover, when G-Rc, G-Rb1 and G-Rd were used as the substrates, BG-1 showed different hydrolytic activity from those of BG-2 and BG-3. These results demonstrate that β-glucosidases from *G. lucidum*, *P. baumii*, *E. applanata* and *P. ostreatus* mycelia selectively hydrolyzed α-(1 → 6)-arabinofuranosidic linkage at the C-20 position of G-Rc without attacking any other β-glucosidic and arabinofuranosidic linkages. These results were also similar to those for ginsenoside Rb1-hydrolyzing β-d-glucosidases purified from *Achatina fulica* (Luan et al. 2006), *Cladosporiun fulvum* (Zhao et al. 2009), and ginsenoside α-arabinofuranosidase isolated from fresh ginseng root (Zhang et al. 2002). G-Rb1-hydrolyzing β-glucosidases from *A. fulica* and *C. fulvum* have highly selective hydrolytic activities toward the β-(1 → 6)-glucosidic linkage attached to the C-20 position of PPD-type ginsenosides without any activity toward other glucosidic linkages.

The fruiting body of *A. mellea*, known as honey mushroom, has been used as a health food in various forms and for dietary supplementation (Chen et al. 2015). In traditional Chinese medicine, the fruiting body and mycelia of *A. mellea* have been used for treating a variety of complaints including palsy, headache, hypertension, insomnia, vertigo, neurasthenia, and for neuroprotection (Lung and Chang 2011; Chen et al. 2015). As Fig. 8a shows, the hydrolysis of G-Rc by BG-1 was significantly influenced by the reaction temperature. BG-1 exhibited potent G-Rc hydrolyzing activity from 40 to 60 °C; the optimum temperature for C-Mc formation was between 55 and 60 °C while that for C-K formation from G-Rc was between 45 and 50 °C. Generally, the optimum temperatures for ginsenoside hydrolyzing enzymes from human intestinal bacteria and soil microorganisms are in the range 37–45 °C (Park et al. 2010; Wang et al. 2011; Yang et al. 2015). The optimum temperature range of BG-1 in this study was slightly higher than those in previous studies that reported the optimum temperatures of the ginsenoside hydrolyzing enzymes from microorganisms such as *Aspergillus* sp., *Penicillium* sp., *Trichoderma* sp., *Absidia* sp., and *Bifidobacterium* sp. which were all in the range of 37–50 °C (Park et al. 2010; Yang et al. 2015). The β-glucosidases prepared from the cultured mycelia of white rot fungi such as *Lentinus edodes*, *Grifola fondrosa*, *Polyporus squamosus*, and *Trametes versicolor* exhibited temperature optima between 60 and 70 °C (Mfombep et al. 2013). The optimum pH for C-Mc and C-K formation from G-Rc by BG-1 was between 4.0 and 4.5 and the hydrolytic activity of G-Rc was decreased above pH 5.0. A previous study (Mfombep et al. 2013) reported that the optimum pH range for β-glucosidase activities from the cultured mycelia of white rot fungi was between 3.8 and 5.0. Our result showed that weakly acidic conditions are ideal for the formation of C-Mc and C-K from G-Rc by BG-1. The effect of pH on microbial and enzymatic hydrolysis of PPD-type ginsenosides has been extensively studied with microbial enzymes isolated from various sources. These enzymes for the transformation of PPD-type ginsenosides including G-Rc showed optimal activity in the range pH 4.0–6.0 (Park et al. 2010; Yang et al. 2015). In this study, the conversion ratio of G-Rc into C-K and C-Mc was greatly influenced by the enzyme concentration. When 2.0 mg of G-Rc was incubated with reaction mixture (1 ml) containing 1.6 U of β-glucosidase activity for 96 h at 45 °C, G-Rc was mostly hydrolyzed to C-Mc and C-K, with a conversion yield of 43 and 48%, respectively. Several β-glycosidases with the ability to transform major PPD-type ginsenosides into C-K have been reported. The biotransformation ratios from G-Rb1, G-Rb2 or G-Rc into C-K as the sole metabolite by microbial β-glucosidases from *Paecilomyces bainier*, *Pyrococcus furiosus* or *Terrabacter ginsenosidimutans* were between 77 and 94% (Yang et al. 2015). However, unusually, β-glucosidase from *Sulfolobus acidocaldarius* produced C-Mc from G-Rc, with a conversion yield of 100% (mol/mol) (Noh and Oh 2009).

In conclusion, BG-1, one of β-glucosidases purified from *A. mellea* mycelia, exhibited potent hydrolytic activity toward G-Rc. The optimum conditions for C-Mc and C-K formation from G-Rc were reaction time of 72–96 h and pH 4.0–4.5. The optimum temperature for C-K formation from G-Rc was 45–50 °C, while that for C-Mc formation was between 55 and 60 °C. The pathways for formation of C-Mc and C-K form G-Rc were G-Rc → C-Mc1 → C-Mc and G-Rc → G-Rd → G-F2 → C-K, respectively. C-Mc was also slowly hydrolyzed α-(1 → 6)-arabinofuranosidic linkage at the C-20 site to produce C-K with reaction time prolongation (≥96 h). These results suggest that β-glucosidase (BG-1) purified from *A. mellea* mycelia can be used to efficiently produce more pharmacologically active ginsenosides, C-Mc and C-K from G-Rc under controlled reaction conditions.

## Abbreviations

AMMEP: ammonium sulfate (30–80%) precipitate isolated from *Armillaria mellea* mycelia
HPLC: high performance liquid chromatography
UPLC/Q-TOF-MS: ultra performance liquid chromatography/quardruple-time of flight mass spectrometry
NMR: nuclear magnetic resonance
BSA: bovine serum albumin
DEAE: diethylaminoethyl
MUG: 4-methylumbelliferyl-β-d-glucopyranoside
SDS-PAGE: sodium dodecyl sulfate–polyacrylamide gel electrophoresis
TLC: thin layer chromatography
PPD: protopanaxadiol
PPT: protopanaxatriol
*p*NP: *p*-nitrophenyl
KACC: Korean Agricultural Culture Collection
ESI: electrospray ionization
UV: ultraviolet
